# Problématique de la prise en charge du cancer de l'enfant: expérience du rétinoblastome à Lubumbashi (RD Congo) et importance du diagnostic précoce

**DOI:** 10.11604/pamj.2013.14.64.2342

**Published:** 2013-02-14

**Authors:** Gray Kanteng A Wakamb, Gayllord Mutoke Nkashama, Robert Lukam Ba Mbuli, Gaby Chenge Borasisi, Julien Ilunga Nikulu

**Affiliations:** 1 Service de pédiatrie, Cliniques Universitaires de Lubumbashi, RDC; 2 Centre ophtalmologique, Cliniques Universitaires de Lubumbashi, RDC; 3 Service d'anatomo-pathologie, Cliniques Universitaires de Lubumbashi, RDC

**Keywords:** Prise en charge, rétinoblastome, enfant, diagnostic précoce, management, retinoblastoma, childhood, early diagnosis

## Aux editeurs du Journal Panafricain de Médecine

Le cancer est une maladie rare chez l'enfant; il constitue seulement 2 à 3% de l'ensemble des cancers recensés [1]. Cependant, même dans les pays développés où des avancées notables en matière de dépistage et de prise en charge sont effectuées, le cancer de l'enfant demeure encore un grand défi de la médecine actuelle, car il est généralement greffé d'une lourde mortalité. En France par exemple, le cancer était en 2009 la 2^ème^ cause de décès chez l'enfant de moins de 15 ans après les accidents [2]. Or jusqu’à 80% des enfants atteints de cancer vivent dans les pays à ressources limitées [3].

Peu d’études épidémiologiques sont rapportées en Afrique, la plupart des pays ne possédant pas de registres nationaux d'enregistrement annuel. Quelques études hospitalières donnent néanmoins une idée des statistiques. Kimpambudi au Congo-Brazzaville en 2000 a évalué à 6,4 cas pour 100.000 habitants l'incidence du cancer chez l'enfant [4].

Le type histologique du cancer de l'enfant est bien particulier puisque les carcinomes, qui représentent l′immense majorité des tumeurs de l′adulte, sont pratiquement absents chez l′enfant. Chez lui, outre les leucémies et lymphomes qui représentent 45% des affections malignes, on trouve essentiellement des tumeurs dites embryonnaires et ensuite seulement les sarcomes plus proches des tumeurs de l'adulte [5]. Ainsi, contrairement au cancer de l'adulte, le cancer de l'enfant a la particularité de se développer en général très rapidement, mais également d’être plus chimiosensible. Il existe donc une réelle opportunité de survie si le diagnostic est posé précocement et le traitement entrepris à temps. L'expérience rapportée des cas de rétinoblastomes traités conjointement à l'unité d'oncologie pédiatrique et au centre d'ophtalmologie des Cliniques universitaires de Lubumbashi illustre bien cette réalité. Le rétinoblastome est une tumeur relativement facile à diagnostiquer. Les signes d'appels sont la leucocorie, un strabisme; et plus tardivement une buphtalmie ou une exophtalmie. Des symptômes que les parents peuvent apprendre à reconnaitre dès le départ. La confirmation du diagnostic est souvent faite au simple fond d''il. Diagnostiqué au stade précoce (intraoculaire), plus de 90% des cas sont guéris dans les pays développés [6]. Le pronostic est réservé au stade tardif (stade extraoculaire: atteintes tumorales orbitaires, extensions ganglionnaires prétragiennes et cervicales ou atteintes métastatiques).

**Figure 1:**
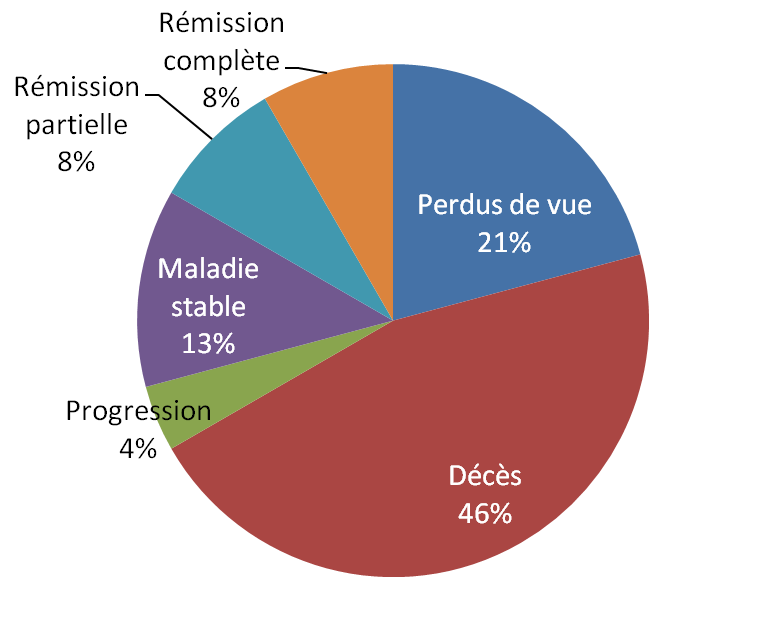
Issue des rétinoblastomes

Selon le Tableau 1, incluant 24 cas de rétinoblastomes aux Cliniques universitaires de Lubumbashi traités depuis 5 ans (entre 2008 et 2012) il ressort que 91,8% des patients ont consulté tardivement, soit ayant été référés en retard ou ayant erré auprès des praticiens de médecine traditionnelle. Ainsi l'issue est souvent catastrophique, avec un nombre important des décès et des perdus de vue (Figure 1).

**Tableau 1:** Fréquence et délai de diagnostic des rétinoblastomes

Délai du diagnostic	Effectif	%
Stade précoce (intraoculaire)	2	8,3
Stade tardif (extraoculaire)	22	91,7
**Total**	**24**	**100**

La difficulté liée au diagnostic tardif du cancer de l'enfant est commune à beaucoup de pays africains. Une étude indiquait qu'au Malawi, qui compte 6,5 millions d′enfants de moins de 15 ans âge, environ 900 cas de cancer infantile sont prévisibles chaque année (sur la base des taux d′incidence européens). Moins de 300 cas par an sont enregistrés, ce qui laisse les deux tiers des enfants portés disparus [3]. Les résultats étaient similaires dans une étude sur l′état des soins en oncologie pédiatrique dans 10 pays à bas et moyen revenus, dont deux pays d′Afrique subsaharienne (la Tanzanie et le Sénégal) [7].

Il est un fait que la RD Congo est un pays à ressources limitées. Néanmoins les mesures pouvant améliorer la prise en charge et infléchir la mortalité liée au cancer de l'enfant sont accessibles. Il existe des atouts qui peuvent déjà être exploités. A Lubumbashi il existe une unité d'oncologie pédiatrique ‘uvrant aux Cliniques Universitaires de Lubumbashi. Elle est parrainée par le Groupe Franco-Africain d'Oncologie Pédiatrique qui la supplée en médicaments anticancéreux. Elle bénéficie d'une pluridisciplinarité avec les services d'imagerie, de chirurgie, d'ophtalmologie et d'anatomo-pathologie.

Le défi pour le rétinoblastome et pour tous les cancers de l'enfant dans le contexte de notre milieu, reste le rôle de la sensibilisation et de l’éducation des masses. C'est probablement ce sur quoi doivent encore oeuvrer les prestataires des soins et la politique nationale de santé.

En l'occurrence, dans le cas du rétinoblastome, un transfert immédiat des malades présentant les signes d'appel au centre de référence est indispensable. La systématisation de l'examen du fond d''il chez ces malades permettrait de cibler précocement la population à risque.

## Conclusion

Avec l'appui de plusieurs organismes dont l'Organisation Mondiale du Cancer, l'unité d'oncologie pédiatrique et le centre ophtalmologique des Cliniques Universitaires de Lubumbashi ont lancé depuis le début de 2013 un programme de sensibilisation des masses sur le rétinoblastome. Un projet similaire a été lancé depuis 2011 à Bamako. Selon une étude les résultats sur une campagne de sensibilisation au Honduras ont permis de réduire sensiblement le retard de consultation [8]. L'objectif à court terme est de faire connaitre les signes du rétinoblastome à la population et de favoriser le diagnostic précoce. A long terme l'objectif est d’étendre le processus de vulgarisation à la plupart des cancers de l'enfant afin de réduire la mortalité.
